# Direct RT-qPCR detection of SARS-CoV-2 RNA from patient nasopharyngeal swabs without an RNA extraction step

**DOI:** 10.1371/journal.pbio.3000896

**Published:** 2020-10-02

**Authors:** Emily A. Bruce, Meei-Li Huang, Garrett A. Perchetti, Scott Tighe, Pheobe Laaguiby, Jessica J. Hoffman, Diana L. Gerrard, Arun K. Nalla, Yulun Wei, Alexander L. Greninger, Sean A. Diehl, David J. Shirley, Debra G. B. Leonard, Christopher D. Huston, Beth D. Kirkpatrick, Julie A. Dragon, Jessica W. Crothers, Keith R. Jerome, Jason W. Botten

**Affiliations:** 1 Division of Immunobiology, Department of Medicine, Robert Larner, M.D. College of Medicine, University of Vermont, Burlington, Vermont, United States of America; 2 Virology Division, Department of Laboratory Medicine and Pathology, University of Washington, Seattle, Washington, United States of America; 3 Vermont Integrative Genomics Resource, Robert Larner, M.D. College of Medicine, University of Vermont, Burlington, Vermont, United States of America; 4 Department of Pathology and Laboratory Medicine, University of Vermont Medical Center, Burlington, Vermont, United States of America; 5 Vaccine and Infectious Disease Division, Fred Hutchinson Cancer Research Center, Seattle, Washington, United States of America; 6 Department of Microbiology and Molecular Genetics, Robert Larner, M.D. College of Medicine, University of Vermont, Burlington, Vermont, United States of America; 7 Vaccine Testing Center, Robert Larner, M.D. College of Medicine, University of Vermont, Burlington, Vermont, United States of America; 8 Data Science Division, IXIS, Burlington, Vermont, United States of America; 9 Department of Pathology and Laboratory Medicine, Robert Larner, M.D. College of Medicine, University of Vermont, Burlington, Vermont, United States of America; 10 University of Vermont Health Network, Burlington, Vermont, United States of America; 11 Division of Infectious Disease, Department of Medicine, University of Vermont Medical Center, Burlington, Vermont, United States of America; Georgia Institute of Technology, UNITED STATES

## Abstract

The ongoing COVID-19 pandemic has created an unprecedented need for rapid diagnostic testing. The World Health Organization (WHO) recommends a standard assay that includes an RNA extraction step from a nasopharyngeal (NP) swab followed by reverse transcription–quantitative polymerase chain reaction (RT-qPCR) to detect the purified SARS-CoV-2 RNA. The current global shortage of RNA extraction kits has caused a severe bottleneck to COVID-19 testing. The goal of this study was to determine whether SARS-CoV-2 RNA could be detected from NP samples via a direct RT-qPCR assay that omits the RNA extraction step altogether. The direct RT-qPCR approach correctly identified 92% of a reference set of blinded NP samples (*n* = 155) demonstrated to be positive for SARS-CoV-2 RNA by traditional clinical diagnostic RT-qPCR that included an RNA extraction. Importantly, the direct method had sufficient sensitivity to reliably detect those patients with viral loads that correlate with the presence of infectious virus. Thus, this strategy has the potential to ease supply choke points to substantially expand COVID-19 testing and screening capacity and should be applicable throughout the world.

## Abstract 2

A major bottleneck to RT-PCR-based COVID-19 testing is the RNA extraction step. This study shows that a “direct RT-PCR” approach that omits the RNA extraction step can reliably detect SARS-CoV-2 RNA in nasopharyngeal swabs from individuals most likely to be infectious.

## Results/Discussion

The ongoing COVID-19 pandemic has put exceptional strain on public health laboratories, hospital laboratories, and commercial laboratories as they attempt to keep up with demands for SARS-CoV-2 testing. The current diagnostic testing methods recommended by the Centers for Disease Control and Prevention (CDC) in the United States and the World Health Organization (WHO) are traditional reverse transcription–quantitative polymerase chain reaction (RT-qPCR) assays that require 2 steps: an RNA extraction from patient nasopharyngeal (NP) swab material, followed by RT-qPCR amplification of the extracted RNA to detect viral RNA [1–3]. A major bottleneck to widespread SARS-CoV-2 testing lies at the RNA extraction step. By mid-March of 2020 many RNA extraction kits were completely sold out, and reagents for manual kits as well as reagents and supplies for the larger automated instruments remain extremely limited, with uncertain supply chains. While substitution of other RNA extraction kits [4–6] is possible, they too are in limited supply. RNA extraction represents a choke point not only due to shortages of the required reagents, but also due to the cost of the extraction process, the labor and time required to perform it, and the fact that it is rate limiting compared to the downstream RT-qPCR analysis. It is also worth noting that RNA extraction uses more liquid handling steps than the downstream RT-PCR, with corresponding requirements for consumables and reagents. While recent Emergency Use Authorizations have been approved by the US Food and Drug Administration for commercial extraction-free diagnostic tests, these are proprietary systems; protocols that work with the open-source RT-qPCR assay developed by the WHO are needed. To address this issue, we tested the performance of a RT-qPCR approach eliminating the RNA extraction step altogether, instead directly loading patient swab material into the RT-qPCR mix. Herein, we report that this approach (which we refer to hereafter as “direct RT-qPCR”) correctly identified 92% of samples (*n* = 155) identified as positive for SARS-CoV-2 RNA by conventional RT-qPCR featuring an RNA extraction. This approach has a sensitivity of 95% on samples with a clinical cycle threshold (CT) at or below 32, which corresponds to a viral load of 1.7 × 10^4^ copies/ml. Notably, this is considerably below the threshold at which samples have been shown to contain live virus and are thought to be infectious (10^6^–10^7^ copies/ml) [7,8]. Thus, our results suggest that this streamlined assay can robustly detect potentially infectious individuals and greatly alleviate constraints to COVID-19 testing in many regions of the world.

**Fig 1:**
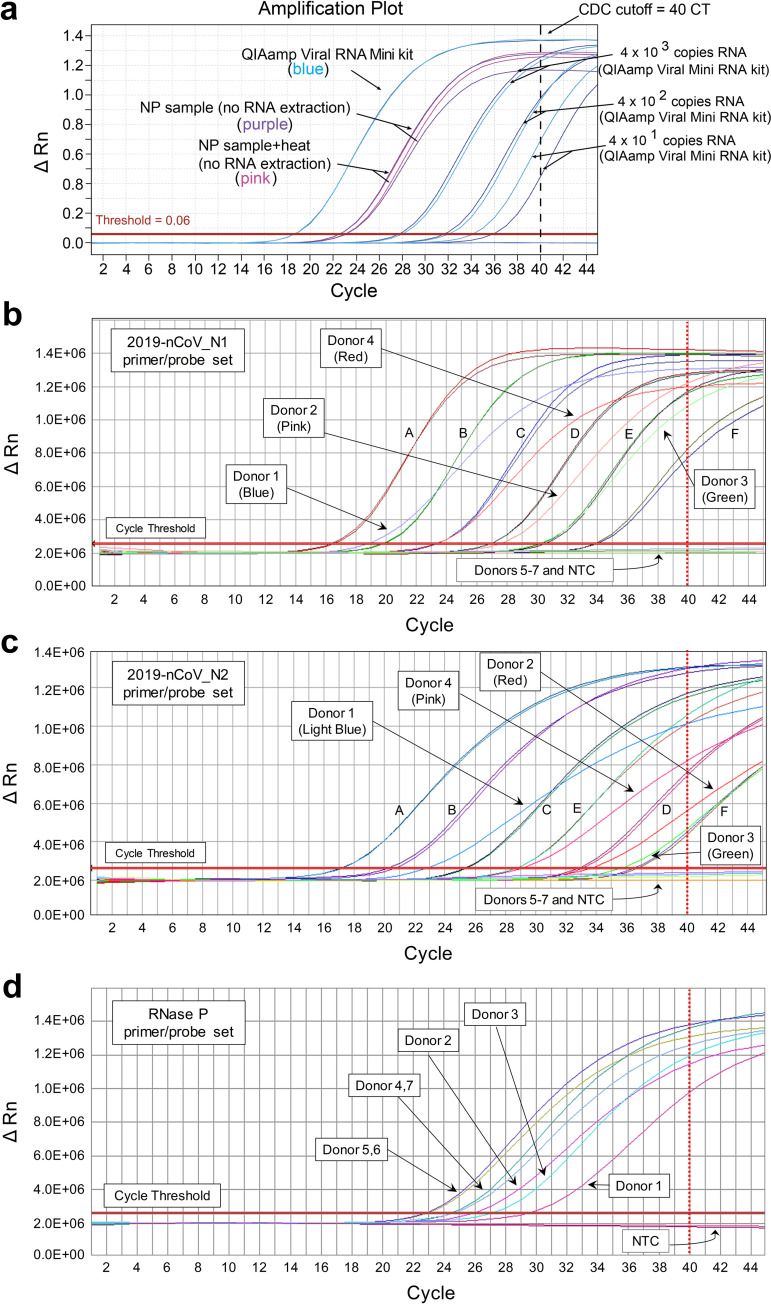
SARS-CoV-2 RNA can be detected from COVID-19 patient NP swabs by RT-qPCR without an RNA extraction step. (A) NP swab diluents from 2 confirmed COVID-19 patients were pooled, and using the 2019-nCoV_N3 primer/probe set, the mixture was either (i) subjected to RNA extraction using the Qiagen QIAamp Viral RNA Mini Kit followed by subsequent testing by RT-qPCR (using the equivalent of 11.3 μl of swab diluent) or (ii) directly added to the RT-qPCR reaction, with or without a preheating step (5 minutes at 70°C, “NP sample + heat”). As a control, the indicated quantities of the CDC 2019-nCoV Positive Control SARS-CoV-2 synthetic RNA were spiked into M6 transport medium, purified using the QIAamp Viral RNA Mini Kit, and screened by RT-qPCR. NP swab samples from 7 additional donors were screened by direct RT-qPCR for SARS-CoV-2 RNA using the 2019-nCoV_N1 primer/probe set (B) or the 2019-nCoV_N2 primer/probe set (C), or for human RNase P RNA using the RNase P primer/probe set (D). NP swab samples from donors 1–4 were previously shown to contain SARS-CoV-2 RNA by standard clinical RT-qPCR, while donors 5–7 were negative. For each primer/probe set, 7 μl (A) or 3 μl (B–D) of NP swab diluent was tested in the RT-qPCR reaction per donor. For the N1 and N2 primer/probe sets, the fully synthetic SARS-CoV-2 RNA Control 2 from Twist Bioscience was loaded at serial 10-fold dilutions (A, 3 × 10^6^ copies; B, 3 × 10^5^ copies; C, 3 × 10^4^ copies; D, 3 × 10^3^ copies; E, 3 × 10^2^ copies; F, 3 × 10^1^ copies) as indicated in (B) and (C). NTC wells were included for each primer/probe set, and each was negative. For (B) and (C), the correlation coefficients (*R*^2^) of the standard curves were 0.999 and 0.995, respectively. The dashed line at cycle 40 in each graph indicates the limit of detection. CDC, Centers for Disease Control and Prevention; CT, cycle threshold; NP, nasopharyngeal; NTC, no template control; RT-qPCR, reverse transcription–quantitative polymerase chain reaction.

We initially conducted a pilot experiment using NP swabs from 2 COVID-19 patients who had previously been verified as having SARS-CoV-2 infection by the Vermont Department of Health Laboratory using the CDC’s recommended RT-qPCR test. The samples were originally collected as NP swabs in 3 ml of M6 viral transport medium (termed diluent hereafter), and were pooled for this analysis (equi-volume). RNA was extracted from 140 μl of the pooled sample using the QIAamp Viral RNA Mini Kit, and purified RNA representing 11.3 μl of the original swab diluent was detected as positive via standard RT-qPCR using the CDC 2019-nCoV_N3 primer/probe set, with a CT of 18.7. In parallel, we added 7 μl of the pooled COVID-19 patient NP swab diluent directly to the RT-qPCR reaction, and found that SARS-CoV-2 RNA was successfully detected in the absence of an RNA extraction step. Compared to the same pooled NP swab diluent extracted with the QIAamp Viral RNA Mini Kit (after adjusting for the quantity of swab diluent added in each case), adding the NP diluent directly into the RT-qPCR reaction resulted in an approximately 4 CT delay in sensitivity (Fig 1A). Preheating the NP diluent for 5 minutes at 70°C prior to RT-qPCR had no impact on viral RNA detection. These results provided proof of principle that successful detection of SARS-CoV-2 RNA from an NP swab sample by RT-qPCR could be done in the absence of an RNA extraction step.

We next sought to validate the direct RT-qPCR approach on additional samples, determine the optimal volume of NP swab diluent for use in the direct RT-qPCR assay, and further address the potential impact of a higher temperature prior heating step on assay sensitivity. NP samples from 3 COVID-19 patients that had previously been shown to be positive for SARS-CoV-2 RNA at high, intermediate, or low copy load by the Department of Laboratory Medicine at the University of Washington (UW) in Seattle were heated or not at 95°C for 10 minutes and then directly loaded into RT-qPCR reactions at a volume of 1, 3, or 5 μl, or subjected to RNA extraction via the Roche MagNA Pure 96 platform prior to loading the equivalent of approximately 20 μl of swab diluent into the RT-qPCR reaction. SARS-CoV-2 RNA could be detected in all 3 viral copy level samples at any of the input volumes by direct RT-qPCR, provided they were heated first (Table 1). Heating appeared to be important for detection of low viral copy samples, presumably because it denatured RNases or inhibitors of the reverse transcriptase and/or PCR enzymes present in the NP diluent and/or enhanced the availability of viral RNA through direct lysis of cells and virions. Addition of less NP diluent led to more sensitive detection of target RNA. The best sensitivity for SARS-CoV-2 detection was achieved when 3 μl of swab diluent was used for direct RT-qPCR (Table 1).

**Table 1:** Detection of SARS-CoV-2 RNA from NP swab diluent by direct RT-qPCR and the impact of heat and loading volume on assay sensitivity.

Sample	Direct RT-qPCR (no RNA extraction)^a^						RT-qPCR^b^ (with RNA extraction)	
	5 μl of swab diluent		3 μl of swab diluent		1 μl of swab diluent		20 μl of diluent equivalent	
	95°C	No heat	95°C	No heat	95°C	No heat	95°C	No heat
NP #1	24.0	26.5	20.7	21.2	20.9	20.8	16.8	15.8
NP #2	28.6	33.6	26.1	25.7	26.4	27.0	22.1	20.3
NP #3	38.2	NEG	33.1	33.7	33.2	33.8	28.5	26.5
UTM	NEG	NEG	NEG	NEG	NEG	NEG	NEG	NEG

NP swab diluent was either heated for 10 minutes at 95°C or not prior to either direct or standard RT-qPCR. NEG indicates that a sample was negative after 40 cycles of qPCR.

^a^The indicated volumes of NP swab diluent were loaded directly into RT-qPCR featuring the 2019-nCoV_N2 primer/probe set, and the resulting CT values of each sample are shown.

^b^Standard RT-qPCR assay. The equivalent of 20 μl of RNA extracted from each NP swab sample was loaded into RT-qPCR featuring the 2019-nCoV_N2 primer/probe set, and the resulting CT values of each sample are shown.

CT, cycle threshold; NEG, negative; NP, nasopharyngeal; RT-qPCR, reverse transcription–quantitative polymerase chain reaction; UTM, universal transport medium.

The CDC RT-qPCR method includes an internal control primer/probe set for detection of human RNase P. To ensure that direct RT-qPCR was able to detect the presence of this gene in NP swab diluent, and to test whether our approach was generalizable to other primer/probe sets, we screened 7 donors (*n* = 4 SARS-CoV-2 positive; *n* = 3 SARS-CoV-2 negative) using the 2019-nCoV_N1, 2019-nCoV_N2, and RNase P primer/probe sets. Both the 2019-nCoV_N1 and 2019-nCoV_N2 primer/probe sets specifically detected SARS-CoV-2 RNA in each sample from COVID-19 patients, but not in samples from the negative donors (Fig 1B and 1C). In addition, RNase P was successfully detected in each donor by direct RT-qPCR (Fig 1D). Collectively, these results confirmed the validity of our direct RT-qPCR approach with additional virus-specific primer/probe sets for detection of SARS-CoV-2 RNA and a primer/probe set to detect the presence of the RNase P gene/mRNA in all samples tested.

**Fig 2:**
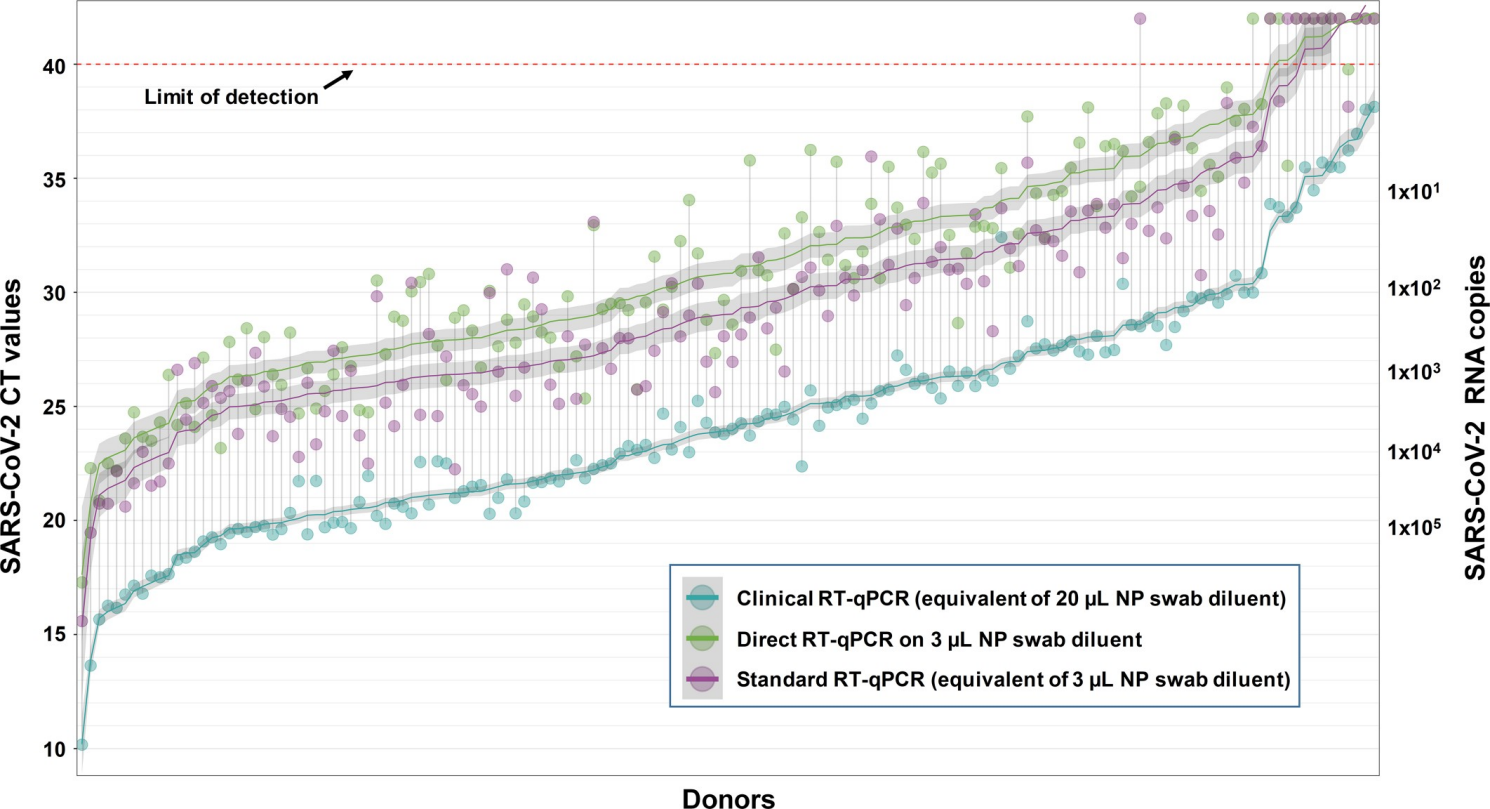
Distribution of CT values from COVID-19 patient NP swabs following direct RT-qPCR versus standard RT-qPCR that included RNA extraction. A total of 150 NP swab samples representing high (CT values less than 20), intermediate (CT values of 20–30), or low (CT values of more than 30) SARS-CoV-2 RNA loads as determined by standard clinical RT-qPCR at the University of Washington in Seattle (aqua circles) were analyzed by the indicated method. All assays used the 2019-nCoV_N2 primer/probe set. Direct RT-qPCR was performed on 3 μl of NP swab diluent after heating for 10 minutes at 95°C (green circles). In parallel, RNA was extracted from 30 μl of NP swab diluent that had been previously heated at 95°C for 10 minutes, and RNA representing 3 μl of the original diluent was used in RT-qPCR (purple circles) to allow a head-to-head comparison with direct RT-qPCR on the same quantity of NP swab diluent. The limit of detection (CT of 40) is denoted with a dashed line. Samples with CT values above this cutoff were considered negative for SARS-CoV-2 RNA. The fitted curves are LOESS (locally estimated scatterplot smoothing)–smoothed CT values, with 95% confidence intervals in gray, against the mean of CT values detected in the clinical RT-qPCR assay with primer sets N1 and N2. Samples are ordered by the latter mean. The full dataset for this experiment and controls are provided in S1 Table. CT, cycle threshold; NP, nasopharyngeal; RT-qPCR, reverse transcription–quantitative polymerase chain reaction.

To get an accurate view of how omission of the RNA extraction step would perform in a real-world clinical setting, we tested, in a blinded fashion, a panel of 150 NP swabs from COVID-19 patients that encompassed the full range of CT values seen in clinical testing at UW. Samples selected included high (CT less than 20), intermediate (CT of 20–30), or low (CT of more than 30) viral RNA loads by a standard clinical RT-qPCR assay that included RNA extraction (referred to hereafter as a laboratory developed test [LDT]) (S1A Table). The incorporation of a second testing site into our study also helped to evaluate the generalizability of the direct RT-qPCR approach and confirm that our results are not dependent on a single master mix, primer/probe set, or RT-qPCR machine. To address the potential impact of a prior heating step on detection sensitivity, NP swab samples were heated or not for 10 minutes at 95°C prior to use in the downstream RNA extraction and/or direct RT-qPCR. For each NP swab sample, a 3-μl volume of diluent was used for direct RT-qPCR. In parallel, a 30-μl aliquot of each sample was used for RNA extraction and an equivalent of 3 μl of the original swab diluent was used for RT-qPCR, allowing for a one-to-one comparison with the direct RT-qPCR method. To control for inhibitors of the reverse transcriptase or PCR enzymes and/or RNase activity in the swab diluent, an aliquot of each swab diluent was spiked with 4 × 10^4^ copies of EXO control RNA for subsequent detection with an EXO primer/probe set. While it has been reported that the CDC RNase P primers have the potential to amplify both genomic DNA as well as mRNA (as both primers are in the same exon), the EXO spike-in control utilized in the UW LDT provides a built-in control for successful RT-PCR amplification from a known RNA template [9]. Inhibition was noted in only 2 samples of 240 tested, 1 negative sample and 1 positive sample tested by the direct approach (S1B and S2B Tables). Preheating NP swab samples prior to direct RT-qPCR enhanced assay sensitivity (138/150 samples positive with preheating, compared with 126/150 without; Table 2). In contrast to the results with the direct RT-qPCR assay, preheating samples reduced sensitivity when using the Roche MagNA Pure 96 RNA extraction system. Extraction of RNA prior to RT-qPCR did not enhance detection of SARS-CoV-2-positive samples when an equivalent amount of diluent was screened by direct RT-qPCR (138 of 150 samples were detected by each approach), suggesting that much of the benefit of kit-based RNA extraction comes from concentrating the sample material, allowing more to be loaded into the RT-qPCR reaction. Of the 12 samples not detected by direct RT-qPCR, 11 were from donors that had extremely low loads of viral RNA as originally determined in the clinical test, with CT values ranging from 33 to 38 (Fig 2; S1A Table).

**Table 2:** Detection sensitivity of direct RT-qPCR versus standard RT-qPCR on NP swabs containing a range of SARS-CoV-2 viral RNA loads.

Viral RNA load^a^	Direct RT-qPCR (3 μl of swab diluent)^b^		Standard RT-qPCR (3 μl of swab diluent equivalent)^c^		Standard RT-qPCR (20 μl of swab diluent equivalent)^d^	
	95°C	No heat	95°C	No heat	95°C	No heat
High (CT < 20)	30/30 (100%)	30/30 (100%)	30/30 (100%)	30/30 (100%)	16/16 (100%)	16/16 (100%)
Intermediate (CT 20–30)	102/103 (99%)	94/103 (91%)	102/103 (99%)	103/103 (100%)	74/74 (100%)	74/74 (100%)
Low (CT > 30)	6/17 (35%)	2/17 (12%)	6/17 (35%)	10/17 (59%)	6/10 (60%)	8/10 (80%)
Total	138/150 (92%)	126/150 (84%)	138/150 (92%)	143/150 (95%)	96/100 (96%)	98/100 (98%)

NP swab diluent was either heated for 10 minutes at 95°C or not prior to either direct RT-qPCR or RNA extraction followed by standard RT-qPCR.

^a^CT values determined by clinical RT-qPCR at the University of Washington in Seattle using the equivalent of 20 μl of RNA extracted from an NP swab. The 2019-nCoV_N2 primer/probe set was used for the RT-qPCR reactions.

^b^The indicated volume of NP swab diluent was loaded directly into RT-qPCR featuring the 2019-nCoV_N2 primer/probe set.

^c^RNA was extracted from 30 μl of NP swab diluent and the equivalent of 3 μl of NP swab diluent was loaded into RT-qPCR featuring the 2019-nCoV_N2 primer/probe set.

^d^RNA was extracted from 200 μl of NP swab diluent and the equivalent of 20 μl of NP swab diluent was loaded into RT-qPCR featuring the 2019-nCoV_N2 primer/probe set.

CT, cycle threshold; NP, nasopharyngeal; RT-qPCR, reverse transcription–quantitative polymerase chain reaction.

**Fig 3:**
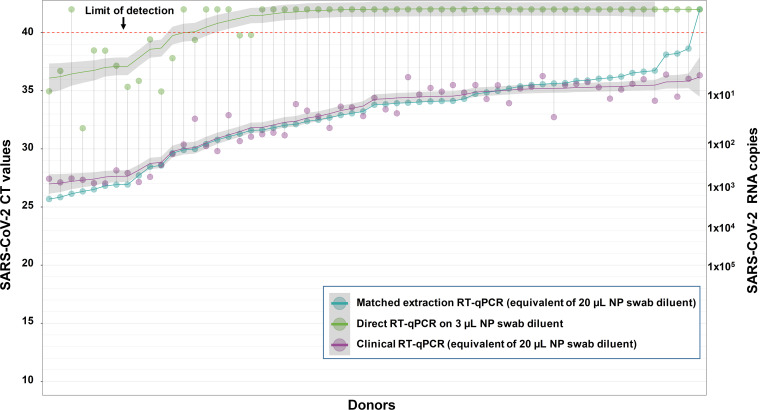
Limit of detection of direct RT-qPCR approach. A total of 60 NP swab samples representing low loads (CT of 27–36) of SARS-CoV-2 RNA as determined by standard clinical RT-qPCR at UW in Seattle (purple circles) were analyzed by the indicated method. All assays used the 2019-nCoV_N2 primer/probe set. Direct RT-qPCR was performed on 3 μl of NP swab diluent after heating for 10 minutes at 95°C (green circles). In parallel, RNA was newly extracted from 200 μl of NP swab diluent (aqua circles) and processed with the UW LDT to control for the effect of freeze/thaw cycles. The limit of detection (CT of 40) is denoted with a red dashed line. Samples with CT values above this cutoff were considered negative for SARS-CoV-2 RNA. The fitted curves are LOESS (locally estimated scatterplot smoothing)–smoothed CT values, with 95% confidence intervals in gray, against the CT values detected in the 200-μl freshly extracted RT-qPCR assay with primer set N2. Samples are ordered by the CT value of freshly extracted 200-μl LDT samples. The full dataset for this experiment and controls are provided in S2 Table. CT, cycle threshold; LDT, laboratory developed test; NP, nasopharyngeal; RT-qPCR, reverse transcription–quantitative polymerase chain reaction; UW, University of Washington.

To more precisely determine the limit of detection of the direct RT-qPCR assay, we tested an additional 60 samples with original clinical CT values between 27 and 36. We specifically screened a large number of patient samples with low viral loads in order to test the limits of the direct assay, although in real-world testing, patients with these CT values represent a minority of overall clinical samples tested. We found the limit of detection (CT value at which 95% of known positives are correctly identified) for the direct approach to be a clinical CT of 32, which corresponds to approximately 50 RNA copies/reaction, or 1.7 × 10^4^ copies/ml of patient sample as measured by the UW clinical LDT (Fig 3; S2A Table). Recent studies suggest that infectious virus can only be isolated from NP swabs that contain at least 10^6^–10^7^ copies of viral RNA/ml [7,8]. To directly relate this to our assay, 10^6^ copies of viral RNA/ml of NP swab diluent is equal to 3 × 10^3^ copies in a 3-μl reaction, which equates to a CT of approximately 25.5 in the direct RT-qPCR assay. Thus, the direct RT-qPCR method described here would have sufficient sensitivity to detect individuals most likely to be infectious.

To determine the specificity of the direct approach we also tested 30 samples known to be SARS-CoV-2 negative by the UW LDT. All 30 samples were negative for SARS-CoV-2 RNA by the direct approach, and 29/30 successfully amplified the EXO spike-in control (S2A and S2B Table). From this result it appears that in rare cases inhibitors in patient samples can prevent amplification of the RT-PCR reaction, but it is worth noting that the EXO internal control failed to amplify only twice out of 240 samples tested in this study (S1B and S2B Tables). The addition of a spike-in control or the use of a set of primers designed to unambiguously detect a human mRNA could provide an important control to rule out false negatives that result from RT-PCR inhibition, though this phenomenon appears to occur only rarely. Collectively, these results demonstrate that the direct RT-qPCR assay described here is capable of reliably detecting SARS-CoV-2 RNA in COVID-19 patients with 100% specificity.

COVID-19 testing demands are currently overwhelming the world’s clinical laboratories. A major choke point is the RNA extraction step, due to both the time and labor for this step and the critical shortage of extraction kits and required reagents. As a means to circumvent these limitations on clinical testing availability, we show here that a direct RT-qPCR approach that omits an RNA extraction step can effectively identify SARS-CoV-2 RNA from NP swabs. When applied to a large collection of clinical NP specimens representative of the range of COVID-19 patients in the state of Washington, the direct RT-qPCR assay correctly identified 92% of the donors screened, with 100% specificity. The only samples missed were those with very low levels of viral RNA, near the limit of detection even when RNA extraction was used. It is likely that the direct assay would detect the most infectious patients, as the ability to isolate live virus from NP swabs correlates with viral loads that are robustly detected here [7,8,10]. In the weeks since we posted our initial results on bioRxiv on March 21, 2020 [4], several groups from around the world have reported similar findings [11–21]. The simplified approach presented here may be especially well suited for general screening of the public and identification of “silent carrier” individuals. A substantial fraction (over 50% in one nursing home surveilled) of SARS-CoV-2-positive cases are asymptomatic or presymptomatic, indicating that a surveillance strategy based on testing only symptomatic individuals would fail to prevent transmission [10]. Notably, the majority of asymptomatic or presymptomatic patients have thus far displayed CT values that the direct RT-qPCR approach would reliably detect.

We propose that the direct RT-qPCR approach described here could be useful in at least 2 settings. First, in regions of the world that have some degree of access to RNA extraction kits to perform the recommended CDC or WHO clinical RT-qPCR test, we envision that this approach could be used as a screening strategy to expand testing capacity to those (such as asymptomatic individuals, nursing home patients, essential workers, and school children) who are not currently receiving tests. As this approach would pick up the majority of cases, including those most infectious as well as presymptomatic or asymptomatic individuals [7,10], we suggest using it as a means to implement additional testing capacity. This would reserve the extraction-based test for medical personnel, hospital inpatients, and others for whom the very highest accuracy is required in order to determine exposure-related transmission precautions. It is important to note that test sensitivity is a function of time post-infection as well as the technical specifications of any given test. As viral loads (and the ability to isolate live virus) decline after the first 5 days of symptomatic disease, a less sensitive testing approach that can be widely applied to people with very mild symptoms on the first day they appear will likely pick up more people than an exquisitely sensitive test delivered days after symptom onset (a situation seen frequently in the United States due to testing capacity shortages) [7,10,22]. Test accuracy is also a function of the prevalence of the disease in the population being tested. With a specificity of 100% and a sensitivity of 92%, the direct RT-PCR approach described here would have a negative predictive value (the probability that a negative is a true negative) ranging from 97.4% to 99.8% with disease prevalences of 25% to 2%, respectively. The positive predictive value (the probability that a positive is a true positive) of this approach is 100% regardless of disease prevalence, given that no false positives were observed in our data.

The testing approach described here could also easily be adopted in more resource limited settings, including large portions of the developing world that at present completely lack access to RNA extraction. A testing approach that uses patient samples directly without RNA extraction would open up perhaps the only viable avenue for widespread testing in these regions. In recent months the scientific community has made enormous strides towards addressing the formidable challenge of providing ample and accessible COVID-19 testing across the globe, and yet the problem is assuredly not solved. An “all-in” approach to testing (which we describe further here [23]) in which the global scientific community continues to develop and validate a range of testing approaches, with the goal of maximizing access and affordability across the world, represents a promising avenue to address this enormous challenge. Beyond the PCR-based approach described here, the current study also provides a reasonable baseline to predict how well other detection platforms (e.g., loop-mediated isothermal amplification [LAMP]) may perform following direct addition of NP swab samples.

## Materials and methods

### Ethical statement

RT-qPCR analysis of clinical samples for this study was approved by the University of Vermont and the University of Washington institutional review boards with a consent waiver. The de-identified samples were determined to be exempt because they were not considered human subjects research due to the quality improvement and public health intent of the work.

### Samples

#### University of Vermont

Clinical NP swabs were collected in 3 ml of M6 transport medium. Patients were confirmed to be negative (3) or positive (6) for SARS-CoV-2 by the Vermont Department of Health using the CDC 2019-Novel Coronavirus (2019-nCoV) Real-Time RT-PCR Diagnostic Panel [1]. Limited quantities of this material were available, so we initially pooled equal amounts of sample from 2 COVID-19 patients (Fig 1A). Subsequently, 4 additional confirmed SARS-CoV-2-positive patients and 4 SARS-CoV-2-negative donors were tested individually for the presence of SARS-CoV-2 RNA or RNase P by direct RT-qPCR of the sample material (Fig 1B–1D).

#### University of Washington

Clinical NP swabs were collected in 3 ml of universal transport medium (UTM). Thirty samples from patients confirmed to be SARS-CoV-2 negative and 210 samples from patients confirmed to be SARS-CoV-2 positive by the University of Washington Medical Laboratory with a range of viral loads (CT values of SARS-CoV-2 with the N2 primer from the original clinical test run at UW ranged from 10.17 to 38.13) and sufficient volume were selected for this study (Figs 2 and 3; Tables 1, 2, S1 and S2).

All patient samples at each testing site were stored at 4°C in between sample collection and transport to the laboratory, and again until clinical testing was carried out, maintaining a standardized sample acquisition and processing protocol.

### RNA extraction

#### University of Vermont

To compare the effect of nucleic acid extraction with direct RT-PCR, RNA from a pooled patient sample was extracted using the QIAamp Viral RNA Mini Kit (Qiagen, Cat. No. 52904) according to the manufacturer’s instructions. A total of 140 μl of pooled NP swab diluent was extracted and eluted in 60 μl; 5 μl (equivalent to 11 μl of original sample) was used for real-time RT-PCR (Fig 1A).

#### University of Washington

To compare the effect of nucleic acid extraction with direct RT-PCR, RNA from 30 μl or 200 μl of patient sample was extracted using the Roche MagNA Pure 96 platform (Roche Lifesciences) and eluted into 50 μl of buffer; 5 μl of RNA (equivalent to 3 μl of original sample for a 30-μl extraction or 20 μl of original sample for a 200-μl extraction) was used for real-time RT-PCR. To monitor RNA recovery and RT-PCR efficiency, 40,000 copies of EXO internal control RNA were added into the lysis buffer and went through the extraction process with each sample (Figs 2 and 3; Tables 1, 2, S1 and S2).

### RT-qPCR detection

#### University of Vermont

In Fig 1A, 7 μl of pooled NP swab diluent (heated to 70°C for 5 minutes, or not), or 5 μl of extracted RNA, was used as input material for the New England Biolabs Luna Universal Probe One-Step RT qPCR Kit (Cat #E3006S, lot #10066679) according to the Integrated DNA Technologies (IDT) recommendation for primers/probes (1.5 μl of primer/probe per reaction) using primer set N3 from IDT’s 2019-nCoV CDC Emergency Use Authorization Kits (20-μl reaction). Subsequently, in Fig 1B–1D, 3 μl of NP swab diluent was used as input material for the Thermo Fisher TaqPath 1-Step RT-qPCR Master Mix, CG (Cat #A15299) using primer sets N1, N2, and RP (human RNase P) from IDT’s 2019-nCoV CDC Emergency Use Authorization Kits (20-μl reaction). Known copies of positive control RNA (CDC 2019-nCoV Positive Control in vitro transcribed RNA for Fig 1A; fully synthetic SARS-CoV-2 RNA Control 2 [Cat# MN908947.3, Twist Bioscience] for Fig 1B and 1C) were used to compare viral RNA quantities in patient samples. Each patient sample, as well as no template control (water as well as M6 transport medium), was run in duplicate; the synthetic RNA standard was run in triplicate. The initial experiment was run on an ABI QuantStudio 6 Flex (Fig 1A), while subsequent experiments were run on an ABI 7500 Fast (Fig 1B–1D). Reagent preparations were performed in a PCR-free clean room equipped with PCR workstations (AirClean Systems). Clinical sample manipulations (RNA extractions or direct addition of NP swab diluent to RT-qPCR plates) were done in a Class IIA Biosafety Cabinet using BSL-2 precautions. RT-qPCR plates were placed directly in biosafety waste at the conclusion of each run.

#### University of Washington

For Figs 2 and 3 and Tables 1, 2, S1 and S2, for each patient sample, 250 μl was heat treated at 95°C for 10 minutes, or not. For direct RT-PCR, 3 μl of this sample, or 5 μl of extracted RNA, was added directly into the RT-PCR reaction mixture. Each real-time RT-PCR reaction contained 400 nM CDC N2 forward and reverse primers and 100 nM N2 probe. To monitor potential RT-PCR inhibition, each RT-PCR reaction was spiked with 40,000 copies of EXO internal control RNA and EXO primers (100 nM EXO forward and 200 nM EXO reverse) and probes (62.5 nM). Real-time RT-PCR assays were performed using the AgPath-ID One-Step RT-PCR kit (Life Technologies) and an ABI 7500 Real-Time PCR System was used to perform the RT-PCR reactions [24]. For samples that were subjected to RNA extraction, 40,000 copies of EXO RNA was spiked into the lysis buffer. Extraction efficiency for each sample was tracked based on the percentage of the spiked EXO RNA that was detected by RT-qPCR. The primer/probe sequences and RT-qPCR conditions for all methods used are provided in Tables 3 and 4.

**Table 3:** Primer/probe sequences.

Name	Description	Oligonucleotide Sequence (5′ → 3′)
2019-nCoV_N1-F	2019-nCoV_N1 forward primer	5′-GAC CCC AAA ATC AGC GAA AT-3′
2019-nCoV_N1-R	2019-nCoV_N1 reverse primer	5′-TCT GGT TAC TGC CAG TTG AAT CTG-3′
2019-nCoV_N1-P	2019-nCoV_N1 probe	5′-FAM-ACC CCG CAT TAC GTT TGG TGG ACC-BHQ1-3′
2019-nCoV_N2-F	2019-nCoV_N2 forward primer	5′-TTA CAA ACA TTG GCC GCA AA-3′
2019-nCoV_N2-R	2019-nCoV_N2 reverse primer	5′-GCG CGA CAT TCC GAA GAA-3′
2019-nCoV_N2-P	2019-nCoV_N2 probe	5′-FAM-ACA ATT TGC CCC CAG CGC TTC AG-BHQ1-3′
2019-nCoV_N3-F	2019-nCoV_N3 forward primer	5′-GGG AGC CTT GAA TAC ACC AAA A-3′
2019-nCoV_N3-R	2019-nCoV_N3 reverse primer	5′-TGT AGC ACG ATT GCA GCA TTG-3′
2019-nCoV_N3-P	2019-nCoV_N3 probe	5′-FAM-AYC ACA TTG GCA CCC GCA ATC CTG-BHQ1-3′
RP-F	RNase P forward primer	5′-AGA TTT GGA CCT GCG AGC G-3′
RP-R	RNase P reverse primer	5′-GAG CGG CTG TCT CCA CAA GT-3′
RP-P	RNase P probe	5′-FAM-TTC TGA CCT GAA GGC TCT GCG CG-BHQ1-3′
EXO-F	EXO forward primer	5′-GGC GGA AGA ACA GCT ATT GC-3′
EXO-R	EXO reverse primer	5′-GGA ACC TAA GAC AAG TGT GTT TAT GG-3′
EXO-P	EXO probe	5′-VIC-AAC GCC ATC GCA CAA T-MGB-3′

**Table 4:** RT-qPCR conditions.

Kit and reaction step	Temperature	Time	Cycles
**NEB Luna Universal Probe One-Step RT-qPCR Kit**			
RT reaction	55°C	10 minutes	1
	95°C	1 minute	
qPCR reaction	95°C	15 seconds	45
	60°C	1 minute	
**Thermo Fisher TaqPath 1-Step RT-qPCR Master Mix, CG**			
RT reaction	25°C	2 minutes	1
	50°C	15 minutes	
	95°C	2 minutes	
qPCR reaction	95°C	15 seconds	45
	55°C	30 seconds	
**AgPath-ID One-Step RT-PCR kit**			
RT reaction	48°C	10 minutes	1
	95°C	10 minutes	
qPCR reaction	95°C	15 seconds	40
	60°C	45 seconds	

NEB, New England Biolabs; RT-qPCR, reverse transcription–quantitative polymerase chain reaction.

### Study design

For both the University of Vermont and UW, sample selection (including information regarding CT values) and RNA extraction/RT-PCR of samples were performed by separate individuals; the person running the assays was blinded to the original clinical CT value of the samples.

## Supporting information

S1 Table **A)** CT values from COVID-19 patient NP swabs following direct RT-qPCR versus standard RT-qPCR for SARS-CoV-2 RNA detection that included RNA extraction. A total of 150 NP swab samples representing high (CT values less than 20), intermediate (CT values of 20–30), or low (CT values of more than 30) SARS-CoV-2 RNA loads as determined by clinical RT-qPCR at UW in Seattle (labeled “original clinical RT-qPCR”) were analyzed by the indicated methods. Direct RT-qPCR was performed on 3 µl of NP swab diluent after heating for 10 minutes at 95°C or not. In parallel, RNA was extracted from 30 µl of NP swab diluent that had been previously heated at 95°C for 10 minutes or not, and RNA representing 3 µl of the original diluent was used in RT-qPCR to allow a head-to-head comparison with direct RT-qPCR on the same quantity of NP swab diluent. For selected samples (*n* = 100), RNA was also extracted from 200 µl of NP swab diluent (per the UW standard clinical protocol) that had been previously heated at 95°C for 10 minutes or not, and RNA representing 20 µl of the original diluent was used in RT-qPCR. Samples below the limit of detection (CT of 40 or more) are designated “NEG.” Three separate experiments (50 NP swabs per experiment, 150 different samples total) were performed and are indicated. A subset of this data is linked to Fig 2. **B)** CT values from COVID-19 patient NP swabs following direct RT-qPCR versus standard RT-qPCR for EXO RNA that was spiked into the swab diluent. A total of 150 NP swab samples representing high (CT values less than 20), intermediate (CT values of 20–30), or low (CT values of more than 30) SARS-CoV-2 RNA loads as determined by clinical RT-qPCR at UW in Seattle (labeled “original clinical RT-qPCR”) were analyzed by the indicated methods. For the donors indicated, an aliquot of swab diluent was spiked with 4 × 10^4^ copies of EXO control RNA prior to RNA extraction or direct addition of sample to the RT-qPCR reaction for subsequent detection with an EXO primer/probe set. Direct RT-qPCR was performed on 3 µl of NP swab diluent after heating for 10 minutes at 95°C or not. In parallel, RNA was extracted from 30 µl of NP swab diluent that had been previously heated at 95°C for 10 minutes or not, and RNA representing 3 µl of the original diluent was used in RT-qPCR to allow a head-to-head comparison with direct RT-qPCR on the same quantity of NP swab diluent. For selected samples (*n* = 100), RNA was also extracted from 200 µl of NP swab diluent (per the UW standard clinical protocol) that had been previously heated at 95°C for 10 minutes or not, and RNA representing 20 µl of the original diluent was used in RT-qPCR. Samples below the limit of detection (CT of 40 or more) are designated “NEG.” Three separate experiments (50 NP swabs per experiment, 150 different samples total) were performed and are indicated. EXO, EXO primer/probe set; N1, 2019-nCoV_N1 primer/probe set; N2, 2019-nCoV_N2 primer/probe set; nd, not done.
(XLSX)

S2 Table **A)** CT values from COVID-19 patient NP swabs or non-COVID-19 patient NP swabs following direct RT-qPCR versus standard RT-qPCR for SARS-CoV-2 RNA detection that included RNA extraction. A total of 90 NP swab samples representing low SARS-CoV-2 RNA loads (CT values of 27 to 36) or non-detected (i.e., negative) SARS-COV-2 RNA as determined by clinical RT-qPCR at UW in Seattle (labeled “original clinical RT-qPCR”) were analyzed by the indicated methods to establish the limit of detection and specificity of the direct RT-qPCR method. Direct RT-qPCR was performed on 3 µl of NP swab diluent after heating for 10 minutes at 95°C. In parallel, RNA was extracted from 200 µl of NP swab diluent (per the UW standard clinical protocol) that had been previously heated at 95°C, and RNA representing 20 µl of the original diluent was used in RT-qPCR. Samples below the limit of detection (CT of 40 or more) are designated “NEG.” These data are linked to Fig 3. **B)** CT values from COVID-19 patient NP swabs or non-COVID-19 patient NP swabs following direct RT-qPCR versus standard RT-qPCR for EXO RNA that was spiked into the swab diluent. A total of 90 NP swab samples representing low SARS-CoV-2 RNA loads (CT values of 27 to 36) or non-detected (i.e., negative) SARS-COV-2 RNA as determined by clinical RT-qPCR at UW in Seattle were analyzed by the indicated methods. For the donors indicated, an aliquot of swab diluent was spiked with 4 × 10^4^ copies of EXO control RNA prior to RNA extraction or direct addition of sample to the RT-qPCR reaction for subsequent detection with an EXO primer/probe set. Direct RT-qPCR was performed on 3 µl of NP swab diluent after heating for 10 minutes at 95°C. In parallel, RNA was also extracted from 200 µl of NP swab diluent (per the UW standard clinical protocol) that had been previously heated at 95°C for 10 minutes, and RNA representing 20 µl of the original diluent was used in RT-qPCR. Samples below the limit of detection (CT of 40 or more) are designated “NEG.” EXO, EXO primer/probe set; N2, 2019-nCoV_N2 primer/probe set.
(XLSX)

## Abbreviations

CDC: Centers for Disease Control and Prevention
CT: cycle threshold
LDT: laboratory developed test
NP: nasopharyngeal
RT-qPCR: reverse transcription–quantitative polymerase chain reaction
UW: University of Washington
WHO: World Health Organization
